# Tactile perception of randomly rough surfaces

**DOI:** 10.1038/s41598-020-72890-y

**Published:** 2020-09-25

**Authors:** Riad Sahli, Aubin Prot, Anle Wang, Martin H. Müser, Michal Piovarči, Piotr Didyk, Roland Bennewitz

**Affiliations:** 1 INM - Leibniz Institute for New Materials, 66123 Saarbrücken, Germany; 2 Department of Physics, Saarland University, 66123 Saarbrücken, Germany; 3 Department of Materials Science and Engineering, Saarland University, 66123 Saarbrücken, Germany; 4 Cluster of Excellence (MMCI), Saarland Informatics Campus, 66123 Saarbrücken, Germany; 5 Università della Svizzera italiana, 6900 Lugano, Switzerland

**Keywords:** Physiology, Psychology, Materials science, Mathematics and computing, Physics

## Abstract

Most everyday surfaces are randomly rough and self-similar on sufficiently small scales. We investigated the tactile perception of randomly rough surfaces using 3D-printed samples, where the topographic structure and the statistical properties of scale-dependent roughness were varied independently. We found that the tactile perception of similarity between surfaces was dominated by the statistical micro-scale roughness rather than by their topographic resemblance. Participants were able to notice differences in the Hurst roughness exponent of 0.2, or a difference in surface curvature of 0.8 $\hbox {mm}^{-1}$ for surfaces with curvatures between 1 and 3 $\hbox {mm}^{-1}$. In contrast, visual perception of similarity between color-coded images of the surface height was dominated by their topographic resemblance. We conclude that vibration cues from roughness at the length scale of the finger ridge distance distract the participants from including the topography into the judgement of similarity. The interaction between surface asperities and fingertip skin led to higher friction for higher micro-scale roughness. Individual friction data allowed us to construct a psychometric curve which relates similarity decisions to differences in friction. Participants noticed differences in the friction coefficient as small as 0.035 for samples with friction coefficients between 0.34 and 0.45.

## Introduction

Shape, size, and roughness are geometrical factors underlying haptic perception. Shape and size describe the macroscopic form of objects while roughness is a statistical surface property at small length scales which can be felt by touch and seen as haze, but whose geometric details are usually not accessible to human senses. Lederman and Klatzky have described how a repertoire of haptic exploration procedures serves to recognize objects and textures quickly and with high accuracy by touch^1^. Following the contour with a fingertip is a typical procedure to identify shape, while lateral motion of fingertips over the surface is used to detect roughness. Roughness perception is known to follow spatial and temporal cues^2,3^. Modeling mechanical strain gradients in the skin caused by contact with rough surfaces and relating it to the distribution of mechanoreceptors allows for predictive models of roughness perception^4^ and for an understanding of neural coding of touch^5^.

While shape and roughness typically refer to different length scales of the object’s geometry, their haptic perception can become interdependent in some cases. As examples, cubes of the same physical volume are perceived as smaller when their surface is rougher^6^. Surface roughness disrupts the performance in search tasks addressing the shape of objects^7^. The discrimination thresholds in active touch for both shape and roughness perception were found to be higher when the respective discrimination task was unexpected, compared to conditions in which the task was expected^8^. The emphasis of shape versus texture perception also depends on the exploration procedures which were applied^9^.

Roughness and surfaces texture also play a key role in the tactile perception of materials^10,11^. Based on an overview of studies on haptic material perception, Okamoto et al. suggested to distinguish between macro and fine roughness based on the different perceptual mechanisms, namely the lower spatial resolution of slowly adapting mechanoreceptors and the higher density of structures required to stimulate fast adapting mechanoreceptors^12^.

Here, we are interested in the tactile perception of randomly rough surfaces, which are self-similar on small scales. Statistical self-similarity of roughness is frequently observed for natural and technological surfaces from geological to Ångstrom length scales^13–15^. Self-similarity means that the statistical surface properties would look the same to the eye in a microscope that magnifies the height by a factor *m* and the lateral dimensions by $m^H$. The Hurst roughness exponent *H*, which is usually greater than zero but less than one, thus states how much the surface’s ruggedness changes when height and lateral dimension are increased with the same magnification. Our surfaces are generated by an algorithm starting from a set of random numbers and are realized by 3D printing (see details on surface definition in the Methods section). While independently generated surfaces with identical *H* have, on average, the same scale-dependent ruggedness, the sequence of local maxima and minima is completely uncorrelated. In contrast, surfaces generated with different *H* but with identical random numbers differ in how roughness changes with the magnification but have a strong topographic resemblance with almost perfect coincidence of local maxima and minima. Relevance and implications of this description of randomly rough surfaces in contact mechanics, adhesion, and friction have been introduced by Persson with examples for the determination of the roughness exponent *H*^14^. For example, the visible topography of road asphalt is determined by the largest stone particles, while the dissipation underlying tire friction is determined by the self-affine roughness at smaller length scale^16^. Note that the self-similarity of roughness across length scales can also be described as fractal dimension $D_f=3-H$ of a self-affine topography^14^.

**Figure 1:**
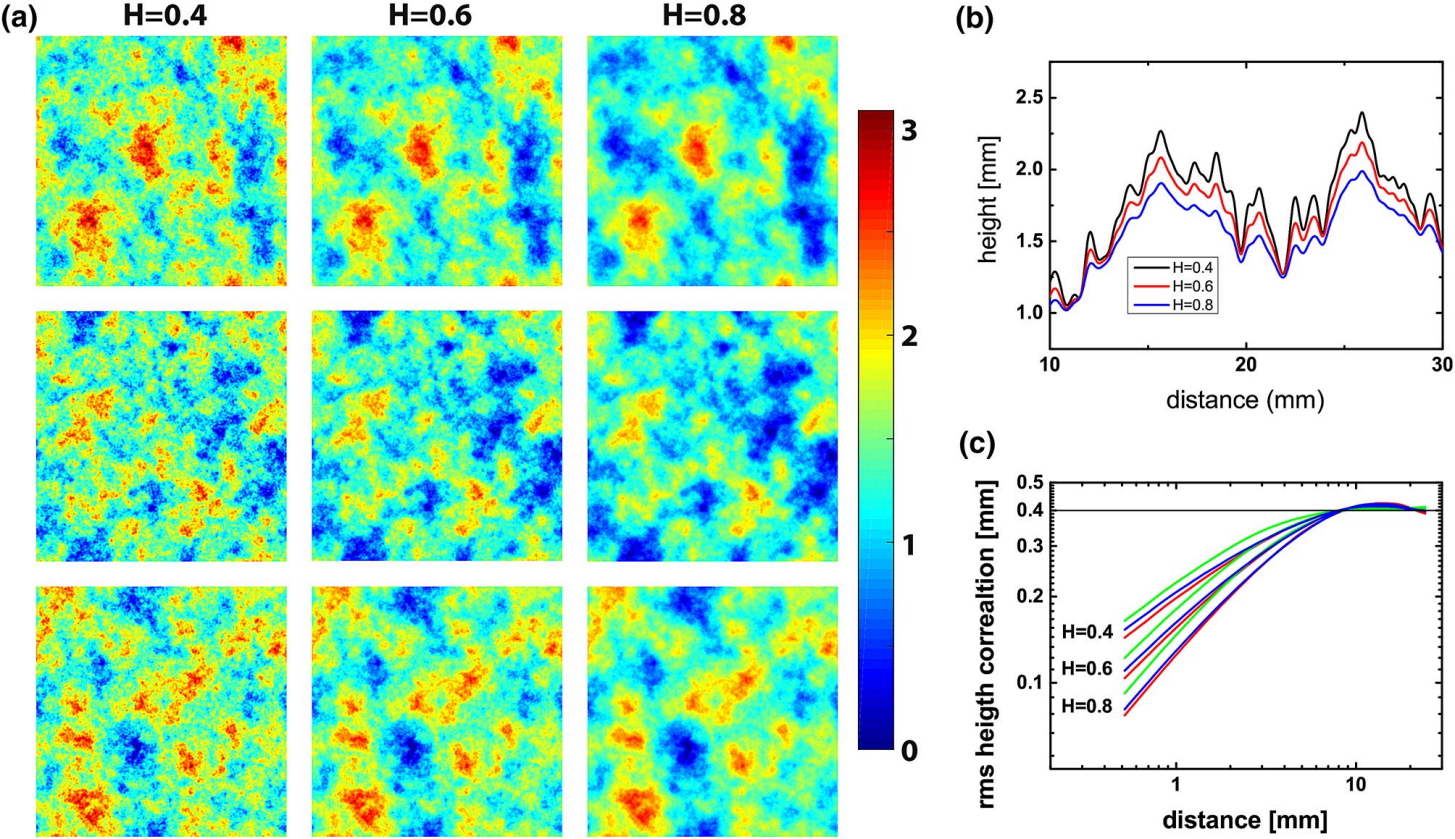
(**a**) Color-coded representation of the height of the nine samples, blue indicates valleys and red crests. Each row shows surfaces with the same topographic structure and Hurst roughness exponents of $H=0.4$, $H=0.6$, and $H=0.8$. Close inspection of the images reveals more spatial detail for lower Hurst exponents, that is a higher roughness amplitude at small length scales. The color scale bar refers to height in mm. (**b**) Cross section with height details for the first 20 mm of the upper edge of the top three images in (**a**). While there is a strong resemblance between the topographic profiles, the fine structure has lower amplitude for higher Hurst exponents. (**c**) Height correlation values for different distances report the rms roughness at different length scales.

Representations of randomly rough surfaces used in our study are given in Fig. 1a, where the surface height is coded as color. Each row of images shows surfaces with strong topographic resemblance, each column shows surfaces with the same Hurst exponent defining roughness towards smaller length scale. The resemblance of topographic features and the decrease in small-scale roughness for higher Hurst exponents is visualized in the cross-sections in Fig. 1b). Regarding the tactile perception of these surfaces, we wondered to what extent the isotropic topographies of randomly rough surfaces are recognized as shape. On the other hand, can humans discriminate the statistical dimension of micro-scale roughness in touch? We used the computational definition of surfaces to vary topographic resemblance and the statistical dimension of microscale roughness independently in order to explore the relative salience of topography and roughness in tactile perception.

## Results

Employing additive manufacturing, we produced a matrix of $$3\times 3$$ samples of $$50\times 50$$$$\hbox {mm}^2$$, where each triple of samples had a strong topographic resemblance, i.e. almost identical locations of maxima and minima. Each triple of samples with this same topographic structure differed in the Hurst roughness exponent *H*. A high Hurst exponent indicates a smooth topography, a lower Hurst exponent a more rugged topography towards small length scales. Figure 1 shows data for the nine surfaces, each three with the same topographic structure but with Hurst roughness exponents *H* of 0.4, 0.6, and 0.8. Mathematically, the topographic structure is defined by the phase relation between harmonic contributions to the height function. The (rms) height correlation decays as power law towards smaller distances with an exponent $$H$$^17^, as shown in Fig. 1c. A higher Hurst exponent corresponds to a faster decay of roughness towards small length scales, resulting in a significantly smaller micro-roughness at length scales of 1 mm and below. The plateau at large distances in Fig. 1c reports the overall roughness of the surfaces. It was set to 0.4 mm in order to keep the overall rms roughness of all samples the same. The plateau’s limits of 7 mm and 25 mm in distance indicate the characteristic lateral extension of larger surface features with equal characteristic height, independent of the Hurst roughness exponent. Our algorithmic definition of randomly rough surfaces provides us with the unique opportunity to vary topographic structure and Hurst roughness exponent independently of each other and thus to evaluate their respective roles in tactile perception. Details on definition and preparation of surfaces are provided in the Methods section.

In our psychophysical experiments, we asked participants to judge the similarity between samples. Similarity is the basis of attributing an example to a category, and is thus essential in the perception and categorization of materials. In the description of the task, we did not state ’with respect to what’ the similarity was to be judged, since we were interested in the perceptional focus of participants. We understand similarity comparisons as a process, in which the ’respects’ for similarity are established in the comparison context^18^. In a study of covert attention in perception by active touch, Metzger et al. told participants only after tactile exploration if the task was discriminating roughness or shape^8^. They found that expectations regarding the task influenced the respective discrimination threshold. The objective of our study was to quantify the relative importance of topography or shape and Hurst exponent or micro-scale roughness in the perception of randomly rough surfaces. We therefore investigated perceived similarity in a forced choice scheme with two alternatives and one reference in order to minimize bias in the decisions^4,19–21^. We will discuss below how our implicit question about similarity still allows to estimate just noticeable differences in friction or micro-roughness.

**Figure 2:**
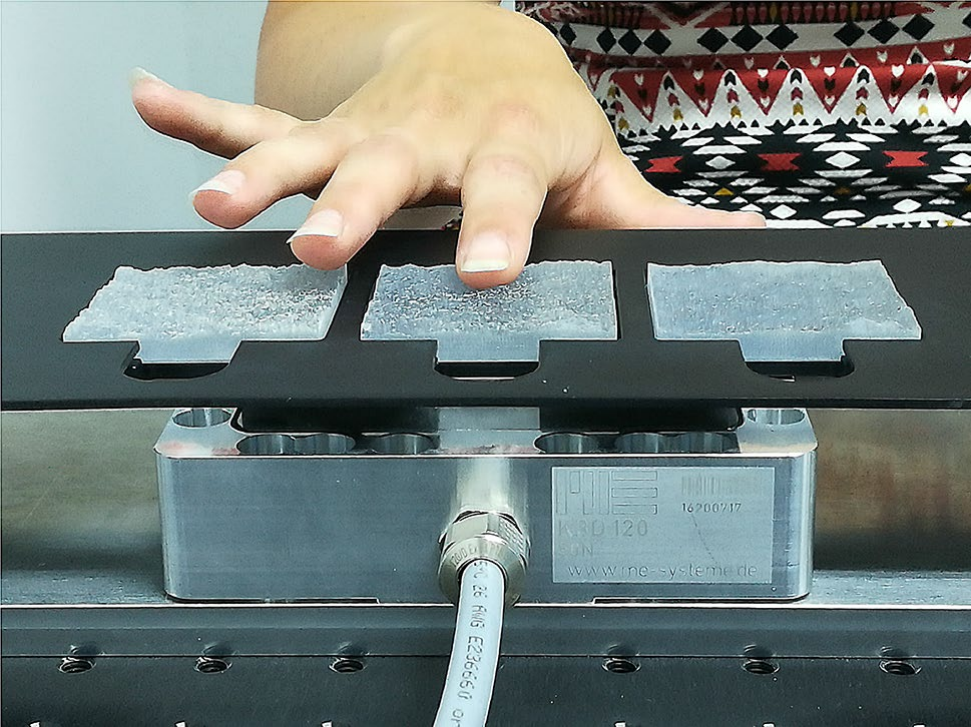
Photograph of the tactile exploration experiment with three of the 3D printed samples mounted on top of the force sensor.

### Perceived similarity of samples with different topography and roughness

Participants compared triples of samples by visual inspection of the images (Experiment 1) and by tactile exploration of the 3D-printed samples through tapping touch (Experiment 2) as well as through sliding touch (Experiment 3) with their index fingertip (see Fig. 2). For details of the experiments, see the section on Methods. Sliding touch in circular motion with the fingertip is both “contour following” and “lateral motion”, the two exploration procedures attributed to the perception of shape and roughness^1^. Participants were asked if “the left or the right sample is/feels more similar to the middle reference.” The list of all decisions was analyzed by means of ordinal multidimensional scaling analysis (MDS analysis), also referred to as non-metric MDS^21^. Readers not familiar with non-metric MDS analysis can find an instructive example in the method section.

**Figure 3:**
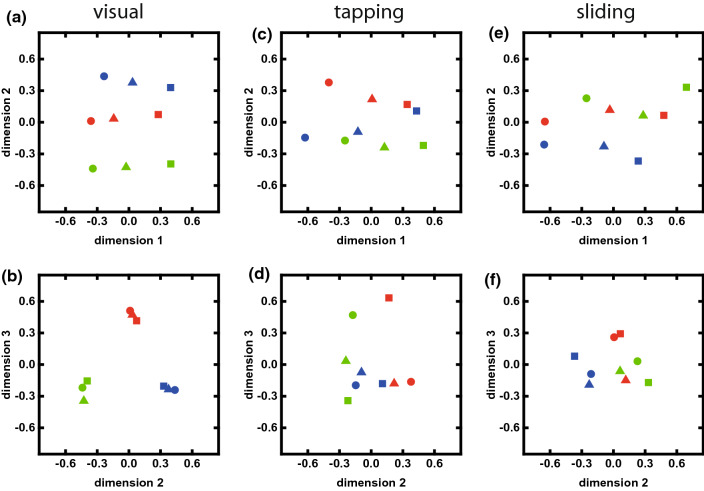
Results of the ordinal three-dimensional scaling analysis of participants’ decisions on perceived similarity. Data points represent surfaces, with colors representing one of three topographic structures and symbols the small-scale roughness (square $H=0.4$, triangle $H=0.6$, circle $H=0.8$). (**a**, **b**) Similarity in visual perception (Experiment 1). (**c**, **d**) Similarity in tactile perception through tapping touch (Experiment 2). (**e**, **f**) Similarity in tactile perception through sliding touch (Experiment 3).

The resulting maps (Fig. 3) display data points representing the nine samples. The distance between data points reproduces the perceived order of similarity by the participants as much as possible: samples perceived as more similar are located closer to each other on the maps. The dimensions of the maps can be thought of as dimensions of perception, which can be linked to relevant characteristics of the samples. We chose three dimensions for the MDS analysis to accommodate the parameters of the sample structure. Two dimensions allow to position the data points representing the three topographic structures at arbitrary distance to each other, the third dimension offers a linear independent axis for the Hurst roughness exponent. The choice of three dimensions is justified by a cross-validation method which was introduced for a similar experimental design in Ref.^21^. Details are provided in the Methods section. Results of ordinal MDS analysis are invariant under rotation, translation, and linear scaling. For the presentation in Fig. 3, the data points have been rotated such that the order of data points along *dimension 1* reproduces best the order of Hurst roughness exponents. We followed the procedure for this *Procrustes analysis* as suggested in Ref.^11^.

We will begin with results of visual inspection of images in Experiment 1. Figure 3a presents a projection onto *dimension 1* and *dimension 2* of the results of a three-dimensional scaling analysis. The nine data points are ordered in an almost regular matrix: Positions along *dimension 1* reproduce the ordering of the Hurst roughness exponent. Distances in *dimension 2* reproduce the difference of topographic structures. In their evaluation of similarity between of images, participants have thus respected both the topographic resemblance and the roughness decay at small length scales. Figure 3b presents a projection of the same results onto *dimension 2* and *dimension 3*. Three clusters of data points lie in an almost equilateral triangle. Each cluster of data points represents images of samples with the same topographic structure, i.e. images in one row in Fig. 1a. In this projection, the average distance of data points for samples with different topography is 8.6 times larger than the average distance of data points within each cluster. We conclude that the topography played a decisive role in the perception of similarity and that the perceptional distance is the same for each pair of the three structures. *Dimension 1* of the MDS results represents the order of Hurst roughness exponents, *dimension 2* and *dimension 3* provide a map of the pairwise dissimilarity of the topographic structures.

The results for tactile exploration by tapping touch (Experiment 2) are presented in Fig. 3c,d. The ordering of data points along *dimension 1* again follows the order of the Hurst roughness exponent, but with more apparent scatter. The order of data points in *dimension 2* still suggests some differentiation of topographic structures, but the projection onto *dimension 2* and *dimension 3* in Fig. 3d reveals that there is no clear separation of data points in clusters of equal topography. In this projection, the average distance between data points for surfaces with different topography is 1.5 times that for surface with equal topography. We conclude that tactile exploration by tapping touch allows to differentiate the Hurst roughness exponent of the surfaces, but leads to only a weak perception of similarity or dissimilarity between surfaces with the same or different topographic structure.

Results for tactile exploration by sliding touch (Experiment 3) are given in Fig. 3e,f. Similar to the results for tapping touch, the MDS analysis reveals a differentiation of the Hurst roughness exponent, but only weak perception of similarity between surface of strong topographic resemblance. In Fig. 3f, the average distance between data points for surfaces with different topography is 1.4 times that for surface with equal topography.

Note that the spread of values along *dimension 1* increases from visual to tapping and to sliding exploration (Fig. 3)a,c,d), while the spread of values over *dimension 2* and *dimension 3* decreases (Fig. 3b,d,f), indicating that the focus in the perception of similarity shifts from the topography to the Hurst roughness exponent.

Quantitative results for the rate of agreement between participants’ decisions on perceived similarity and the samples’ structure are given in Table 1 for decisions between samples of either the same Hurst roughness exponent or the same topographic structure. We discuss these results in order of decreasing rates of agreement. In visual perception (Experiment 1), almost 100 % of decisions recognized that one of the samples had the same topography as the reference, while the other was different. In tactile perception upon tapping touch (Experiment 2) and sliding touch (Experiment 3), 75 % and 78.4 % of decisions recognized that one sample had the same Hurst roughness exponent as the reference. These percentages are at or above 75 %, the standard threshold for a noticeable difference in a two-alternative forced choice (2AFC) experiment. We conclude that the difference between topographic structures is noticeable in visual perception and that a difference of 0.2 in the Hurst roughness exponent is noticeable in tactile perception. This conclusion is drawn for randomly rough surfaces with the given spectral ranges, where topographic features with similar height extend at the scale of the fingertip and where the micro-roughness varies at the scale of the finger ridges.

A fraction of 66.7 % of decisions recognize one topographic structure being equal with the reference after tapping exploration of samples. Similarly, 65.7% of decisions recognize one Hurst roughness exponent being equal to the reference after visual inspection of the representative images. These percentages are below the threshold for a noticeable difference between the two samples, but they still indicate that the topography plays a significant role in the perception of similarity based on tactile exploration by tapping (statistical significance value $p = 0.024$) and that micro-roughness plays a significant role in the visual perception of similarity between the images ($p = 0.018$). No significant influence of the topography was found for similarity perception in sliding touch.

**Table 1:** Percentage of decisions which confirm perceived similarity between the reference and a sample which the same Hurst roughness exponent *H* or the same topographic structure as the reference, while the other sample has a different Hurst roughness exponent or topography.

Decision for the ...	... when choosing between ...	Visual (%)	Tapping (%)	Sliding (%)
Same topographic structure as reference	Two samples of the same *H*	97.2	65.7	56.0
Same *H* as reference	Two samples of the same topographic structure	66.7	75.0	78.4

In order to exclude any bias, only decisions between two samples of the same topographic structure or Hurst roughness exponent, respectively, are counted.

### Perceived similarity between samples of different surface curvature and friction

While the overall rms roughness of all samples is 0.4 mm, the micro-roughness at small length scales varies not only between samples with different Hurst exponent, but also between samples with the same Hurst roughness exponent but different topography. These differences in the height correlation can be observed for small distances in Fig. 1c. Roughness asperities are important for tactile perception. Mechanical bending determines the local strain energy density in the skin and thus the neural activity of the mechanoreceptors with highest spatial resolution^4,5,22^. Skin bending is induced by surface curvature upon touch. Similarly, a tactile sensitivity to shear strain will respond to surface curvature in skin contacts^23^. We therefore calculated the rms curvature of all surfaces (Table 2) as a quantification of micro-roughness using an online tool^24^ which is based on definitions introduced in Ref.^25^. The rms surface curvature is linearly correlated to the height correlation at a distance of 0.75 mm as well as to the rms slope of the surface (see Table 2). The role of surface curvature in the perception of similarity between samples is evaluated by plotting *dimension 1* of the MDS results, which was aligned with the Hurst roughness exponent, versus the rms curvature.

**Table 2:**
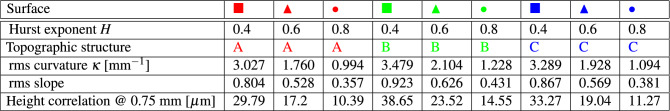
Parameters for the nine surfaces.

The surface curvature $\kappa$, the rms slope, and the height correlation at a distance of 0.75 mm have been evaluated using an online tool^24^.

**Figure 4:**
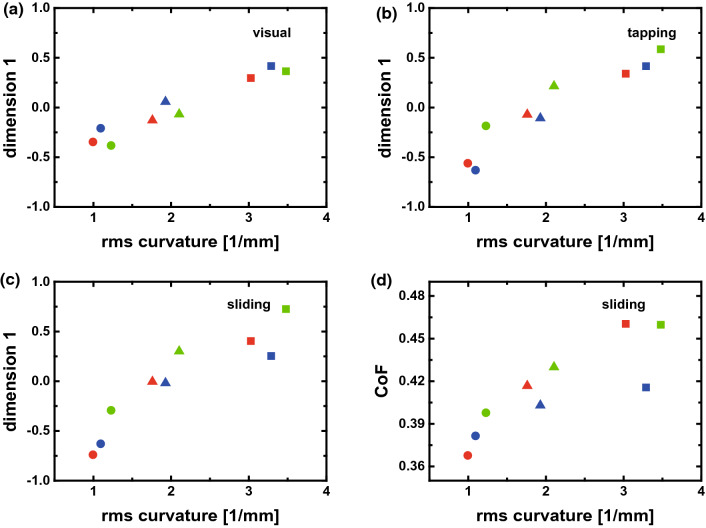
*Dimension 1* of the MDS results plotted versus the root-mean-square (rms) curvature of the surfaces for (**a**) visual, (**b**) tapping touch, and (**c**) sliding touch perception. (**d**) Average coefficient of friction (CoF) plotted versus the rms surface curvature. The coefficient of friction is averaged for each sample over all trials of all participants. Data points represent surfaces, with colors representing one of three topographic structures and symbols the small-scale roughness (square $H=0.4$, triangle $H=0.6$, circle $H=0.8$).

Figure 4a reports a relation between *dimension 1* and the rms curvature for visual perception which reproduces the ordering according to the Hurst roughness exponent. An almost linear relation is found for the case of tactile exploration by tapping (Fig. 4b). A systematic deviation from linearity is observed for the data representing exploration of the different surfaces by sliding touch (Fig. 4c). In *Experiment 3* we recorded the coefficient of friction for the sliding fingertip in all trials. The average coefficient of friction for each sample is plotted as function of the rms surface curvature in Fig. 4d. The strong similarity between the characteristics in Fig. 4c,d suggests that *dimension 1* of tactile perception is best identified with friction when the fingertip slides over the samples.

The nine different values of surface curvature $\kappa$ allow us to construct a metric $D_{i,j}$ for the dissimilarity between arbitrary pairs of sample *i* and sample *j*, which is inspired by the structure of the MDS results. Perceived dissimilarity is predicted as the Euclidean distance in a space spanned by the difference in curvature $\kappa _i-\kappa _j$ and a perpendicular vector which has the length $\alpha$ if the topographic structures of *i* and *j* are different and has the length zero if the topographies are the same:

$$\begin{aligned} D_{i,j}=\sqrt{\left( \kappa _i - \kappa _j\right) ^2 + \left( \alpha (1-\delta _{i,j})\right) ^2} \end{aligned}$$

The parameter $\alpha$ indicates the relevance of a difference in the topographic structure compared to differences in surface curvature for decisions on perceived similarity. Values for $\alpha$ can be estimated by correlating the metric $D_{i,j}$ with the distances between samples *i* and *j* found by MDS analysis. We have maximized the Pearson correlation coefficient $\rho$ by variation of $\alpha$ and found $\alpha =2.68\,\hbox {mm}^{-1} \, (\rho =0.946)$ for the visual perception experiment, $\alpha =1.22\,\hbox {mm}^{-1} \, (\rho =0.708)$ for tapping touch, and $\alpha =0.97\,\hbox {mm}^{-1} \, (\rho =0.743)$ for sliding touch. We also tested alternative metrics with one free parameter, for example the simple linear combination of surface curvature $\kappa$ and difference in topography $\alpha (1-\delta _{i,j})$. The correlation with MDS distance data was weaker than for the metric $D_{i,j}$ for all three experiments.

The perceived visual similarity of surface representations is dominated by the topographic structure: The dissimilarity between different topographies ($\alpha =2.68\,\hbox {mm}^{-1}$) is larger than the largest difference in surface curvature between samples (2.295 $\hbox {mm}^{-1}$, see Table 2). In tapping and sliding touch, differences in the topography play a much lesser role. They contribute to the perceived dissimilarity only about half compared to the largest difference in surface curvature.

### Psychometric functions

**Figure 5:**
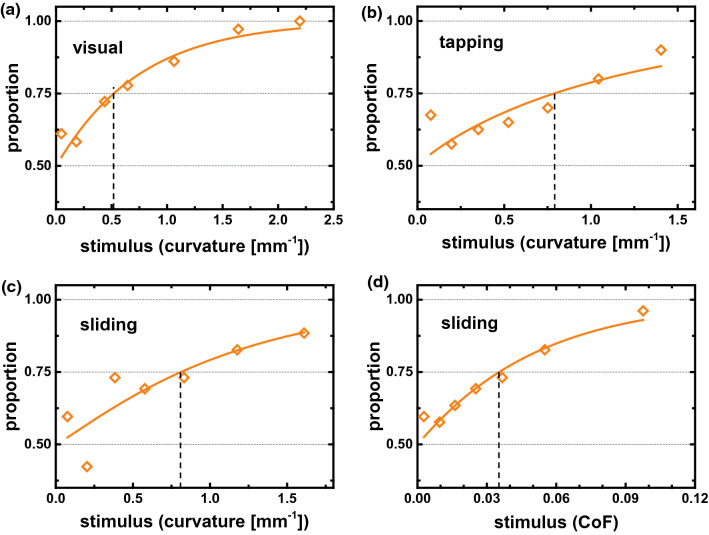
Psychometric curves showing the proportion of expected similarity decisions as function of the stimulus intensity. (**a**–**c**) The stimulus intensity $S=\left| D_{left,ref}-D_{right,ref}\right|$ is calculated as difference in perceived distance as predicted by the metric $D_{i,j}$ between left or right sample and the reference for the three experiments. (**d**) The stimulus intensity $S=\left| \left| \mu _{i}-\mu _{ref}\right| -\left| \mu _{j}-\mu _{ref}\right| \right|$ is calculated from the coefficients of friction measured for each trial and participant. The psychometric functions are modeled by a Weibull sigmoid (**a**) $R=0.93$, (**b**) $R=0.59$ (**c**) $R=0.68$, (**d**) $R=0.93$.

The metric for perceived distance $D_{i,j}$ can be tested on original decision data by calculating psychometric functions. In the two-alternative forced choice experiment, we expect that participants perceive the left sample as more similar to the reference sample compared to the right sample, if $D_{left,ref}<D_{right,ref}$, and vice versa. The stimulus intensity for the decision is then $S=\left| D_{left,ref}-D_{right,ref}\right|$. The psychometric curves in Fig. 5 plot the proportion of decisions as expected versus the stimulus intensity. If the predicted value for the perceived distance between left sample and reference $D_{left,ref}$ is close to the value for right sample and reference $D_{right,ref}$, the stimulus intensity is low and the proportion of correct decisions is 0.5, choosing by chance between the two alternatives. If one sample is predicted to be perceived as much more similar to the reference than the other, the stimulus intensity increases and the proportion of correct decisions approaches 1.

For the visual perception of similarity between surface representations in images (Experiment 1), the psychometric curve has the expected shape. The proportion of correct decisions increases smoothly from 0.5 to 1.0 with increasing stimulus intensity (Fig. 5a, fit to Weibull sigmoid function with $R=0.93$). The data for tapping and sliding touch (Fig. 5b,c) exhibit more scatter with respect to the function ($R=0.59$ and $R=0.68$), in particular at low stimulus intensity. The weaker predictability of participants’ decisions after tactile exploration is in agreement with the less ordered results of the MDS analysis (Fig. 3) and the lower overall success rate of similarity decisions (Table 1).

Similarity decisions might be governed by the topography for trials in which one sample has the same topographic structure as the reference, while the other sample has a different topographic structure. These trials are however equally distributed over all stimulus intensities in all psychometric curves in Fig. 5, thus confirming the adequacy of the metric $D_{i,j}$.

The just noticeable difference in stimulus intensity can be defined as the value at which the proportion of decisions as expected becomes larger than 0.75. For tapping and sliding touch, the participants were able to detect differences of 0.8 $\hbox {mm}^{-1}$, i.e. a difference in surface curvature of 0.8 $\hbox {mm}^{-1}$ was distinguished between samples with the same topographic structure. This just noticeable difference was smaller with 0.5 $\hbox {mm}^{-1}$ for visual perception experiment. Although the physical meaning of surface curvature in the image representations is not obvious, we can state that samples with smaller difference in micro-roughness were distinguished by visual compared to tactile exploration.

For the exploration by sliding touch, we recorded friction coefficient data for each participant and each trial. We tested the conjecture that the samples with the smaller difference in friction coefficient will be perceived as more similar in sliding touch. The psychometric curve was determined for the respective stimulus intensity $S=\left| \left| \mu _{i}-\mu _{ref}\right| -\left| \mu _{j}-\mu _{ref}\right| \right|$ based on the individual coefficient of friction $\mu$ and is shown in (Fig. 5d). The friction-based stimulus intensity produces a smooth psychometric curve which is fitted well by the Weibull sigmoid function ($R=0.93$). The threshold level of 0.75 is reached at a stimulus level of 0.035, i.e. participants were able to detect a difference in friction coefficient of 0.035 between the samples when judging their tactile similarity.

## Discussion

We studied the tactile perception of similarity between randomly rough surfaces, where the topographic structure and the micro-scale roughness were varied independently, and compared the results with those for visual perception of similarity between images representing the height of the surfaces. When judging the similarity, the participants’ focus shifted from the topographic structure in visual perception to the micro-scale roughness in tactile perception. Differences in micro-scale roughness, quantified as mean surface curvature, and the differentness of topographic structures were combined into a dissimilarity metric with good correlation to participants’ similarity judgements. Friction was confirmed as a key tactile dimension in sliding touch and friction data recorded for all participants and trials allowed to successfully construct a psychometric function of similarity decisions.

One objective of our study was to clarify if random surface topographies with structure sizes on the length scale of the fingertip are recognized as shape and contribute to the tactile perception of similarity between surfaces. The results show that in tapping touch (*experiment 2*) the perceived dissimilarity between different random topographies is comparable to a difference of 0.2 in the Hurst roughness exponent, or to a difference in surface curvature of about 1.2 $\hbox {mm}^{-1}$. For sliding touch (*experiment 3*), the difference between topographic structures is even less relevant for the judgment of similarity. No significant influence of the topographic structure on similarity perception was observed for sliding touch. In contrast, the results of *Experiment 1* demonstrate that judgment of similarity for visual inspection of the color-coded representations in Fig. 1a was dominated by the recognition of topographic resemblance. What is the reason for the minor contribution of the actual topography of randomly rough surfaces to the perception of similarity by sliding touch? A circular sliding of the fingertip was imposed as exploration procedure on participants. This procedure appeared to include the “contour following” procedure for shape identification and the “lateral motion” procedure for texture perception suggested by Lederman and Klatzky^1^. On the other hand, any constraint of exploration procedures is known to limit the haptic identification of object shapes^26^. The outstretched finger requires the participant to follow the contour of the surface topography and to sequentially process the respective input^27^. Random isotropic surface profiles do not exhibit easily recognizable features such as periodic structures (for example periodic gratings) or objects with a semantic correspondence (such as the replica of a clover leaf). We suggest that the sequential processing of the random topography with no regular features does not promote an identification of equal topographic structures as being similar. In line with this suggestion, Kuroki et al. reported that 3D-printed surfaces which reproduced photographed motifs of natural structures were not discriminated by different modes of touch^28^. We noted by observation and heard in comments from participants that the tapping touch in *Experiment 2* gave them more opportunity to explore the topography than the fixed sliding motion in circles *Experiment 3*, which explains the greater influence of the differentness of topographic structures on similarity decisions in *Experiment 2*.

In a study on the haptic saliency of different surface features of 3D-printed samples, Metzger et al. found that isotropy and amplitude at smaller spatial scales had strong correlation with the duration of touch of the given surface area in a free exploration task^29^. The high salience of roughness distracting participants in search tasks for shapes had been reported before^7^. These reports suggest to interpret our observations on the dominant role of micro-scale roughness in the tactile perception of similarity as distraction of participants from the perception of topographic structures. Our results also add quantitative support to the hypothesis of Hartcher et al., who invoked micro-scale roughness as an explanation for the surprising observation that tactile roughness discrimination on 3D-printed samples is more sensitive to the printing speed than to the layer height or the build angle in printing^30^.

The mechanical interaction of finger ridges with roughness asperities has been reported to amplify the sensitivity to details of the micro-scale roughness^31–33^. In line with these findings, we suggest that the stimulation of mechanoreceptors by roughness asperities during sliding touch turns the perceptional focus on the roughness at the finger ridge scale in sliding touch and distracts participants from recognition of topographic resemblance at the fingertip scale. We note that the smallest wavelength in our computational definition of the surfaces of 500 $\mu \hbox {m}$, whose amplitude varies most as function of the Hurst exponent *H*, matches the typical distances between papillary ridges of our participants (520 $\mu \hbox {m}\pm$ 80 $\mu \hbox {m}$, extracted from cross-sections along fingerprints).

In a preliminary extension of this study, we were interested if the topographic structure of surfaces starts playing a role in the perception of similarity upon sliding touch (experiment 3) if the question of the task makes explicit reference to the surface profile. We repeated the experiment with 7 new participants and asked whether “the left or right sample is more similar to the middle one with respect to the surface profile. With surface profile, we mean the hills and valleys on the surface. Other words for surface profile may the topography, or the shape of the surface, or the relief of the surface.” With this task, a fraction of 67.7 % similarity decisions recognized that one of the samples had the same topographic structure as the reference, while the other was different. In contrast to the result reported in Table 1, the topography now had a significant influence ($p<0.025$) on the similarity decision after tactile exploration with the sliding fingertip. Despite the explicit question about similarity with respect to the surface profile, a fraction of 69.2 % similarity decisions recognized that one of the samples had the same Hurst roughness exponent *H* as the reference, while the other was different. The influence of *H* on the decisions was still significant ($p<0.025$). We finally repeated the experiment on tactile exploration by the sliding fingertip with 5 expert participants, who knew the structure of samples and the goal of the study. We were interested if the expert participants are able to recognize the same topographic structure within the constraints of the experiment, and added to the task above the sentence “Do not consider the fine roughness.” With this task, 69.6 % of decisions identified the sample with the same topographic structure as similar to the reference while the other sample had a different topography and both sample had the same Hurst exponent *H*. We conclude that the constraints of this experiment, i.e. the sequential tactile exploration in circular movement of the fingertip and the distraction by the fine roughness does not allow to recognize random surface topographies of valleys and hills with a width comparable to that of the fingertip, not even with expert participants. The task not to consider fine roughness was followed by the expert participants, only 57.1 % of similarity decisions recognized if a sample has the same Hurst roughness exponent *H* as the reference while the other had a different *H*, close to a random choice between samples.

We finish this section by a discussion of the role of friction in the tactile perception of similarity between the randomly rough surfaces when explored with a sliding fingertip. Several of ours findings confirm that friction is a relevant tactile dimension: Peculiar characteristics of the dependence of friction on the surface curvature (Fig. 4d) are also observed for *dimension 1* in the MDS analysis of perceived similarity (Fig. 4c). A smooth psychometric function could be constructed for the friction-based stimulus intensity (Fig. 5d). Finally, the topographic structure does not play any significant role in similarity perception with the sliding fingertip (Table 1).

The average friction coefficient increases from 0.35 to 0.45 as the rms surface curvature increases from 1 to 3.5 $\hbox {mm}^{-1}$. It is not a given that friction increases with rms curvature or equivalently with the micro-roughness at small scale. Roughness asperities may lower friction by reducing the real area of contact^34–36^ or increase friction by dissipative deformation when sliding against a viscoelastic counter surface^37–39^ like skin^40,41^ or by elastic interaction with the skin micro-structure^42^. Here we found that friction increases with micro-roughness and that skin deformation by passing asperities causes additional friction. This mechanisms can explain the role of friction as tactile dimension in *Experiment 3*, in particular when assuming that mechanoreceptors in the skin are sensitive to shear strain^23^. A full understanding of fingertip friction on randomly rough surfaces will have to include also the micro-structure of the fingertip skin^42,43^. The quality of friction as predictor for decisions about similarity in *Experiment 3* does not necessarily reveal the mechanisms underlying the similarity perception. Roughness and friction may be coupled as dimensions of tactile perception^44^. However, we suggest that the friction data report on the individual intensity of skin stimulation by roughness asperities in sliding touch for each trial and thus provide the a powerful metric to connect subjective similarity decisions with physical measurements.

## Methods

### Participants

All participants were volunteers working at the INM - Leibniz Institute for New Materials or studying at Saarland University. Participants were naive with respect to the nature of the samples studied and to the goal of the study. They were informed in writing about the procedure and told that they could stop their experiment at any time without giving reason. They gave their informed consent. The study was designed to meet the standards of the Declaration of Helsinki. In particular, risk and burden for all participants were minimized and no members of vulnerable groups were enrolled as participants. The study was approved by the responsible ethics commission in the Department of Human and Business Sciences at Saarland University.

### Surface definition

Nine surfaces (1024 $\times$ 1024 height points, to be realized in a size of 50 $\times$ 50 $\hbox {mm}^2$) were computed with an algorithm written to produce random roughness with well-defined spectral distribution. The design of surfaces starts from a roughness spectrum^17^. The spectrum is composed of a plateau for small wave vectors or large wavelengths, where the corresponding frequency contributions define the macroscopic topography with equal amplitude. For larger wave vectors or smaller distances, the height amplitude decays following a power-law. A larger Hurst exponent *H* leads to smaller amplitudes of the small-scale roughness. The surface is constructed by a superposition of waves, where the amplitudes are picked from the roughness spectrum. The randomness of the surface is introduced by a random phase shift between the superposed waves. We use the same sequence of random numbers to generate each three surfaces with the same topographic structure but varying amplitudes for the superimposed sinusoidal surface undulations. Using a different sequence of random numbers, a different topographic structure was realized, however with the same roughness parameters, i.e. amplitudes. The height values are finally normalized to root-mean-square roughness of 0.4 mm for all surfaces.

The root-mean square curvature $\kappa$ of the surface defined by the height *h*(*x*, *y*) is determined as

$$\begin{aligned} \kappa =\sqrt{\left\langle \left( \frac{\partial ^2 h}{\partial x^2}\right) ^2+\left( \frac{\partial ^2 h}{\partial y^2}\right) ^2\right\rangle _{x,y}}. \end{aligned}$$

### Sample preparation

Samples were printed by a polygraphic printer using a layer thickness of 0.03 mm (RapidObject, Leipzig, Germany). The transparent material VeroClear is similar to polymethyl methacrylate (PMMA) and is heated to only $50^{\circ }\hbox {C}$ during the process. Samples are printed layer by layer and solidified under UV illumination.

### Psychophysical perception experiments

#### Experiment 1: visual perception of false color images

Participants were shown triples of the images (Fig. 1a) on a computer display, each image about $10\times 10\,\hbox {cm}^2$ in size. They were asked to “decide if the left image or the right image is more similar to the middle reference image” and to press one of two respective keys. Participants were told to take their time with the decision. All 252 different combinations of triplets out of the nine samples were offered to participants following a randomly ordered list. Each of the 15 participants (9 male, age 24 to 60) explored 50 triplets typically in a total time of 10-15 minutes.

#### Experiment 2: tactile perception by tapping touch

Each triple of 3D-printed samples was mounted to a holder on a three-axis force transducer (K3D-120, ME Messsysteme, Germany). The experiment was placed on a table and participants were seated at the table with their right shoulder in front of the samples.

Participants were asked to explore the samples by tapping them with the tip of their right index finger. Participants were instructed to follow a predefined sequence of exploration for the triplet of samples, namely “center-left-center-right-center-left-center-right-center”. After finishing the sequence, they had to decide “whether the left or right sample feels more similar to the middle one”, in a forced-choice task. The decision was recorded by the experimenter and the next triple of samples from the randomly ordered list introduced in the description of Experiment 1 was mounted. Each of the 10 participants (5 male, age 22 to 32 explored 28 triplets typically in a total time of 30 minutes.

#### Experiment 3: tactile perception by sliding touch

Experimental setup and procedure were the same as in Experiment 2, except that participants were asked to explore the samples by circular movements with the tip of their right index finger. We asked them to keep the finger straight during movement in order to keep the geometric contact area constant, which would have varied in a circular movement involving bending the finger knuckles. The circular movements had a typical diameter of 3.5 cm and participants chose velocities of 0.5 to 1 circles per second. The friction force $F_f$ and friction coefficient $\mu$ were calculated as

$$\begin{aligned} F_f=\sqrt{F^2_x+F^2_y} \text{ and } \mu =F_f/F_z \end{aligned}$$

where $F_x,F_y$ were the lateral force components and $F_z$ the normal force component. This definition of a linear coefficient $\mu$ is a standard in reporting tactile friction experiments^34–36,38,40,44^. Each of the 13 participants (6 male, age 22 to 53) explored 28 triplets typically in a total time of 45 minutes.

#### Ordinal non-metric scaling analysis

Readers not familiar with multi-dimensional scaling analysis are referred to the introductory text by Borg et al.^45^. As an instructive example for ordinal MDS, we suggest a study were participants are presented with triples of city names A,B, and C out of a list of ten cities from one region or country. Their task is to decide if city A or city B is closer to city C. The decisions of multiple participants on many triples are analyzed by ordinal MDS. The result is a map of the ten cities where the relative distances between cities respect the decisions of participants as far as possible. After rotation, the MDS result probably resembles a geographic map of the cities, albeit with arbitrary scaling. Differences between geographic map and perceptual space can then indicate peculiar features of distance perception between certain cities in participants. In this example, the dimensions of the perceptual space correspond to geographical dimensions. In a study on the perceived similarity in taste of different formulations of a soda drink, the perceptual dimensions could correspond to sweetness and carbon dioxide content.

**Figure 6:**
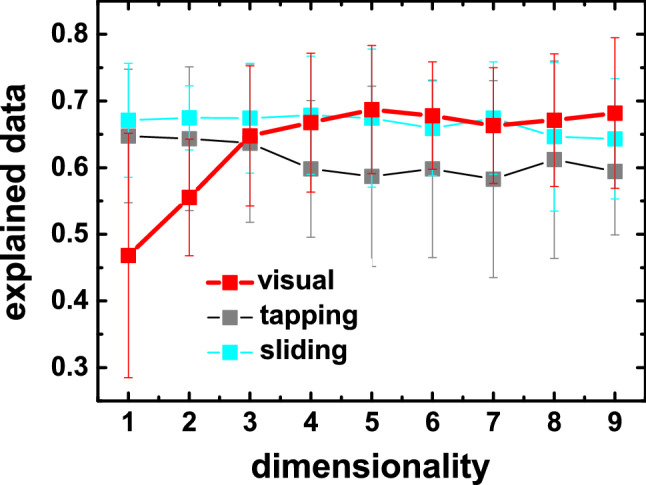
The plot shows the performance of different perceptual spaces in explaining the experimental data as a function of the number of dimensions for all our experiments. The error bars indicate standard deviations computed using tenfold cross-validation.

For all experiments, the perceptual spaces of the ordinal MDS analysis were derived using a formulation provided in Ref.^21^. The method adapts a Bayesian approach^46^ for converting pairwise comparisons into scalar data and finds an *n*-dimensional embedding of the samples by maximizing the probability of observing the experimental data. Using the technique, we analyzed the dimensionality required for capturing our experimental data. More specifically, we performed a 10-fold cross-validation where we recovered a perceptual space using the training set and validated its predictive power on the testing set (Fig. 6). As it can be observed for all experiments, there is no benefit of going beyond three-dimensional space as the performance of more complex spaces in explaining our experimental data remains similar. We have also confirmed that *dimension 1* of the three-dimensional MDS of Experiment 3 (sliding touch) is linearly correlated to the result of the one-dimensional MDS of the same experiment ($\hbox {R}^2=0.996$). Our conclusions for the case of sliding touch thus do not depend on choosing one or three dimensions for the MDS.

To verify the reliability of the recovered perceptual spaces and its robustness to stochastic effects we used bootstrapping^21^. For each experiment, we generated new observations by randomly sampling the experimental data with repetition. Next, we optimized a new perceptual space using the re-sampled data. This procedure was repeated 1000 times. To quantify the statistical dispersion of our data we report the coefficient of variation in pair-wise distances between stimuli in the recovered perceptual spaces. The average coefficient of variation for visual, tapping, and sliding experiments is 0.15, 0.31, and 0.29, respectively. These values suggest that the placements in the recovered visual perceptual space are reliable and while less certain in their judgments, the participants are still able to consistently rate the stimuli during tactile perception studies.

#### Psychometric curves

Psychometric curves are determined for the two sample parameters, the metric $D_{i,j}$ and the coefficient of friction. For psychometric curves, the stimulus intensity data *S* as defined in the text are sorted and divided in bins of equal number of data points. The stimulus intensity value is the average value of each bin. For each bin, the proportion of decisions which meet the prediction based on the respective stimulus is calculated. The sigmoid function fitted to the psychometric curves is a Weibull function:

$$\begin{aligned} P=1-0.5 \cdot \exp {\left( -\left( k\cdot S\right) ^d\right) } \end{aligned}$$

with the proportion of expected decisions *P*, the stimulus intensity *S*, and the Weibull parameters *k* and *d*.
